# Automatic Segmentation of Corpus Callosum in Midsagittal Based on Bayesian Inference Consisting of Sparse Representation Error and Multi-Atlas Voting

**DOI:** 10.3389/fnins.2018.00629

**Published:** 2018-09-11

**Authors:** Gilsoon Park, Kichang Kwak, Sang Won Seo, Jong-Min Lee

**Affiliations:** ^1^Department of Biomedical Engineering, Hanyang University, Seoul, South Korea; ^2^McGill Centre for Integrative Neuroscience, Montreal Neurological Institute, McGill University, Montreal, QC, Canada; ^3^Department of Neurology, Samsung Medical Center, Sungkyunkwan University School of Medicine, Seoul, South Korea

**Keywords:** corpus callosum, segmentation, sparse representation, multi-atlas voting, Bayesian inference

## Abstract

In this paper, we introduce a novel automatic method for Corpus Callosum (CC) in midsagittal plane segmentation. The robust segmentation of CC in midsagittal plane is key role for quantitative study of structural features of CC associated with various neurological disorder such as epilepsy, autism, Alzheimer's disease, and so on. Our approach is based on Bayesian inference using sparse representation and multi-atlas voting which both methods are used in various medical imaging, and show outstanding performance. Prior information in the proposed Bayesian inference is obtained from probability map generated from multi-atlas voting. The probability map contains the information of shape and location of CC of target image. Likelihood in the proposed Bayesian inference is obtained from gamma distribution function, generated from reconstruction errors (or sparse representation error), which are calculated in sparse representation of target patch using foreground dictionary and background dictionary each. Unlike the usual sparse representation method, we added gradient magnitude and gradient direction information to the patches of dictionaries and target, which had better segmentation performance than when not added. We compared three main segmentation results as follow: (1) the joint label fusion (JLF) method which is state-of-art method in multi-atlas voting based segmentation for evaluation of our method; (2) prior information estimated from multi-atlas voting only; (3) likelihood estimated from comparison of the reconstruction errors from sparse representation error only; (4) the proposed Bayesian inference. The methods were evaluated using two data sets of T1-weighted images, which one data set consists of 100 normal young subjects and the other data set consist of 25 normal old subjects and 22 old subjects with heavy drinker. In both data sets, the proposed Bayesian inference method has significantly the best segmentation performance than using each method separately.

## Introduction

The Corpus Callosum (CC), the largest white matter (WM) structure placed beneath the cortex of the human brain, connects homologous cortical areas of the two cerebral hemispheres (Caminiti et al., 2009). It has been shown that many neurological diseases give rise to changes of structural feature of the CC such as size and shape, for example epilepsy (O'Dwyer et al., 2010; Firat et al., 2014), autism (Frazier et al., 2012; Prigge et al., 2013), schizophrenia (Kim et al., 2012; Joshi et al., 2013; Balevich et al., 2015), dyslexia (Casanova et al., 2010; Elnakib et al., 2012), Alzheimer's disease (Zhu et al., 2014; Elahi et al., 2015; Wang et al., 2015) and in the effect of heavy drinking (Bookstein et al., 2002), and smoking (Lin et al., 2013). The CC defined in midsagittal plane (MSP) of the magnetic resonance imaging (MRI) has been generally used because it has relatively clear boundary line and mirrors overall properties of CC (Casanova et al., 2010; O'Dwyer et al., 2010; Joshi et al., 2013; Firat et al., 2014; Zhu et al., 2014; Balevich et al., 2015; Elahi et al., 2015; Wang et al., 2015). Accurate segmentation of CC in MSP is important for the quantitative and qualitative studies of the neurological diseases related to CC.

The manual segmentation of CC is a laborious and time-consuming task. It also has inter-intra variability problem. The more data there is, the more these problems become more serious. For solving the problems resulting from manual segmentation, Various automated methods have been proposed to segment CC (He et al., 2007; Shyu et al., 2012; Içer, 2013; Yeo et al., 2014). Although the general shape of the CC is relatively regular, automated segmentation of CC has been still challenging because the detail shape is dynamic and the fornix, bundle of never fibers located around CC, has exceedingly similar intensity as CC.

Voxel intensity-based segmentation is one of the most popular approaches. Semra Içer proposed the estimated Gaussian mixture model and fuzzy C means from intensity distribution of CC (Içer, 2013). Voxel intensity-based segmentation, however, cannot work well if data has noise or the structures like target structure because it did not consider local information, intensity information of neighborhood voxels in a target voxel. Active Contour Model (ACM) is also popularly used to segment CC. For segmentation of target structure, ACM uses energy consisting of external and internal force. The external force is image features such as gradients and lines and the internal force is the relationship between points of contour. Both the external and internal force develop initial contour to boundary of target structure. He et al. proposed a context-sensitive active contour based on evolving four seed contours, forming interconnected parts made by initial points, into CC boundary line according to each motion law (He et al., 2007). Shyu et al. introduced an ACM based on region-based local intensity with a global density distance (Shyu et al., 2012). Yeo et al. introduced a level-set model based on Bayesian inference, which drew the active contour to the target structure according to image feature and shape prior (Yeo et al., 2014). Although ACM has more robust segmentation performance than the voxel intensity-based segmentation because of considering various information around contour, ACM-based segmentation cannot work well if the initial contour is set far from target structure. Furthermore, it is difficult to segment detailed part of target structure having severe variation because of internal energy.

Recently, the multi-atlas voting method and the patch-based method have been widely used in medical imaging and show good performance in practice (Liu et al., 2012; Tong et al., 2013; Asman et al., 2015; Kim et al., 2015; Sanroma et al., 2015; Zhang et al., 2015; Doshi et al., 2016). The multi-atlas voting method developed from single-atlas -based segmentation. Single-atlas based segmentation estimates a target structure of an unseen image using an atlas image we know the right answer of the target structure (Andreasen et al., 1996; Cuadra et al., 2004). When applied to the target image with heavily variation, the single atlas based segmentation generally shows poor performance due to registration error. Multi-atlas voting based segmentation, which uses multiple atlases instead of just one atlas, has been proposed to overcome this issue. For final segmentation result in multi-atlas voting based segmentation, multi-classifier voting algorithm such as majority voting and STAPLE is required (Warfield et al., 2004). Aljabar et al. suggested atlas selection, where subset atlas appropriate for target image are selected through similarity index between atlas and target image in order to reduce registration error and computational time (Aljabar et al., 2009). The multi-atlas voting based segmentation is still vulnerable to the detailed estimation of the brain structure when the number of atlas is limited and variation of the target image is large. Patch-based segmentation uses abundant neighborhood features (e.g., intensity or gradient) of a target voxel in target image, overcoming lake of local information. Sparse representation, a patch-based technique, has been introduced in various image processing fields such as denosing (Elad and Aharon, 2006), restoration (Mairal et al., 2008), and face recognition (Wright et al., 2009; Yang and Zhang, 2010). The basic concept of sparse representation is that an input target image could be reconstructed as a sparse linear combination of dictionary samples consisting of training images via L1-norm minimization (Donoho, 2006). Sparse representation procedure results in generating sparse coefficients and reconstruction error between the target image and the sparse approximate image (Donoho, 2006). Recently, sparse representation technique has been applied successfully in neuroimaging fields such as volume registration (Kim et al., 2015), hippocampus segmentation (Tong et al., 2013), AD classification (Liu et al., 2012), and brain parcellation (Zhang et al., 2015). Since the segmentation method using sparse representation is based on abundant local information, it has good performance about the detailed part of target structure. But it is ineffective about overall shape in comparison with multi-atlas voting. In this paper, we combined the strengths of multi-atlas voting method and sparse representation method for CC segmentation. The proposed method avoids the disadvantages of the pervious CC segmentation methods and has better performance than multi-atlas voting method and sparse representation method, respectively.

There have been several studies in which the target structure is segmented by combining two type information. Patenaude et al. built a Bayesian framework, consisting of probabilistic relationships between shape and intensity extracted atlas images, to segment subcortical region of target image (Patenaude et al., 2011). Sanroma et al. introduced a novel segmentation method that fused two types of patch-based label fusion approaches, reconstruction-based approaches and classification-based approaches, using matrix completion for segmentation of various brain structures (Sanroma et al., 2015). These methods prove that segmentation method with a good combination of the two types of information can lead to better performance than single type information.

We combined information extracted from sparse representation and multi-atlas voting using Bayesian inference framework. Bayesian inference has been used in various fields for a long time. It effectively combines prior information and likelihood. Prior information can be specified by the user, but usually represents a natural probability. In other words, it calculates the probability that the target belongs to each class based on the experience so far. In this paper, it is extracted from the probability map resulting from multi-atlas voting. Since likelihood is calculated based on certain condition, we estimate the class-conditional probability that agrees with the distribution of reconstruction errors generated from sparse representation in each class. Multi-atlas voting based segmentation has good segmentation performance toward global information such as overall shape and location since atlases effectively catch the global information of the target image using registration. Patch-based segmentation (i.e., sparse representation based segmentation) well catches local information such as detailed shape variation since patches contain relation between target voxel and its neighborhood voxels. We could successfully mix the two types of information, which are global and local generated from multi-atlas voting and sparse representation respectively, in Bayesian inference. To the best of our knowledge, a combined method of sparse representation and multi-atlas voting in Bayesian inference has not been used for segmentation.

In this paper, we proposed a novel Bayesian inference consisting of sparse representation and multi-atlas voting for CC segmentation. The proposed method used the sparse representation error of target image as the likelihood and the probability map of CC generated from multi-atlas voting as a prior information. We compared the performance of the proposed method with those of other related methods as follow: (1) the joint label fusion (JLF) method which is state-of-art method in multi-atlas voting based segmentation for evaluation of our method; (2) prior information estimated from multi-atlas voting only; (3) likelihood estimated from sparse representation error (LESRE) only; (4) the proposed Bayesian inference consisting of sparse representation error as likelihood and probability map of CC as a prior. Finally, we validated robustness and accuracy by applying the proposed method to the two data set, where one consists of young healthy group and the other consists of heavy drinkers that generally have large variability of CC shape (Bookstein et al., 2002).

## Methods

### Data acquisition

We used two different data sets for evaluation (Table 1). The first data set (“OASIS”) was selected from Open Access Series of Imaging Studies (OASIS) database (www.oasis-brain.org), which consists of three or four individual T1-weighted MR brain images of 416 subjects. We randomly selected 100 normal male and female subjects among them (50 males, age range = 18–28 years, mean age = 22.1 years, standard deviation = 2.7 years). The images were acquired 1.5T MRI scanner (Siemens, Erlangen, Germany) using magnetization-prepared rapid gradient sequence [TR = 9.7 ms, TE = 4 ms, slice thickness = 1.25 mm, flip angel = 10, TI = 20 ms, TD = 200 ms, matrix size = 256 × 256 pixels (1 mm × 1 mm)]. All subjects used in this work gave written informed consent and the use of these subjects was approved by the Institutional Review Boards (IRB) of Washington University (www.oasis-brains.org). The details have been described in Marcus et al. (2007) for OASIS20. The second data set (“SMC”) consisted of T1-weighted MR brain images of 47 male subjects included 22 heavy drinkers (age range = 44–80 years, mean age = 64.5 years, standard deviation = 9.1 years). The SMC data set was obtained from Samsung Medical Center of Seoul in South Korea. The Institutional Review Board (IRB) of Samsung Medical Center approved this study. The participants in this study provided written informed consent. The T1-weighted images were acquired 3T MRI scanner (GE Signa, Milwaukee, WI) using spoiled-gradient echo sequence (TR = 30 ms, TE = 7 ms, slice thickness = 1.5 mm, number of excitations = 1, flip angle = 45°, field of view = 22 × 22 cm^2^, matrix size = 256 × 256 pixels). The CC shapes of the SMC data set are more variable due to heavy drinker and old age.

**Table 1:** Demographic information of OASIS data set and SMC data set used in this study.

**Data set (T1-weighted)**	**Number (male)**	**Composition (subject number)**	**Age range**	**Mean age (standard deviation)**
**OASIS**	100 (50)	Normal (100)	18–28 years	22.1 years (2.7 years)
**SMC**	47 (47)	Normal (25) Heavy drinker (22)	44–80 years	64.5 years (9.1 years)

### Pre-processing

**Figure 1:**
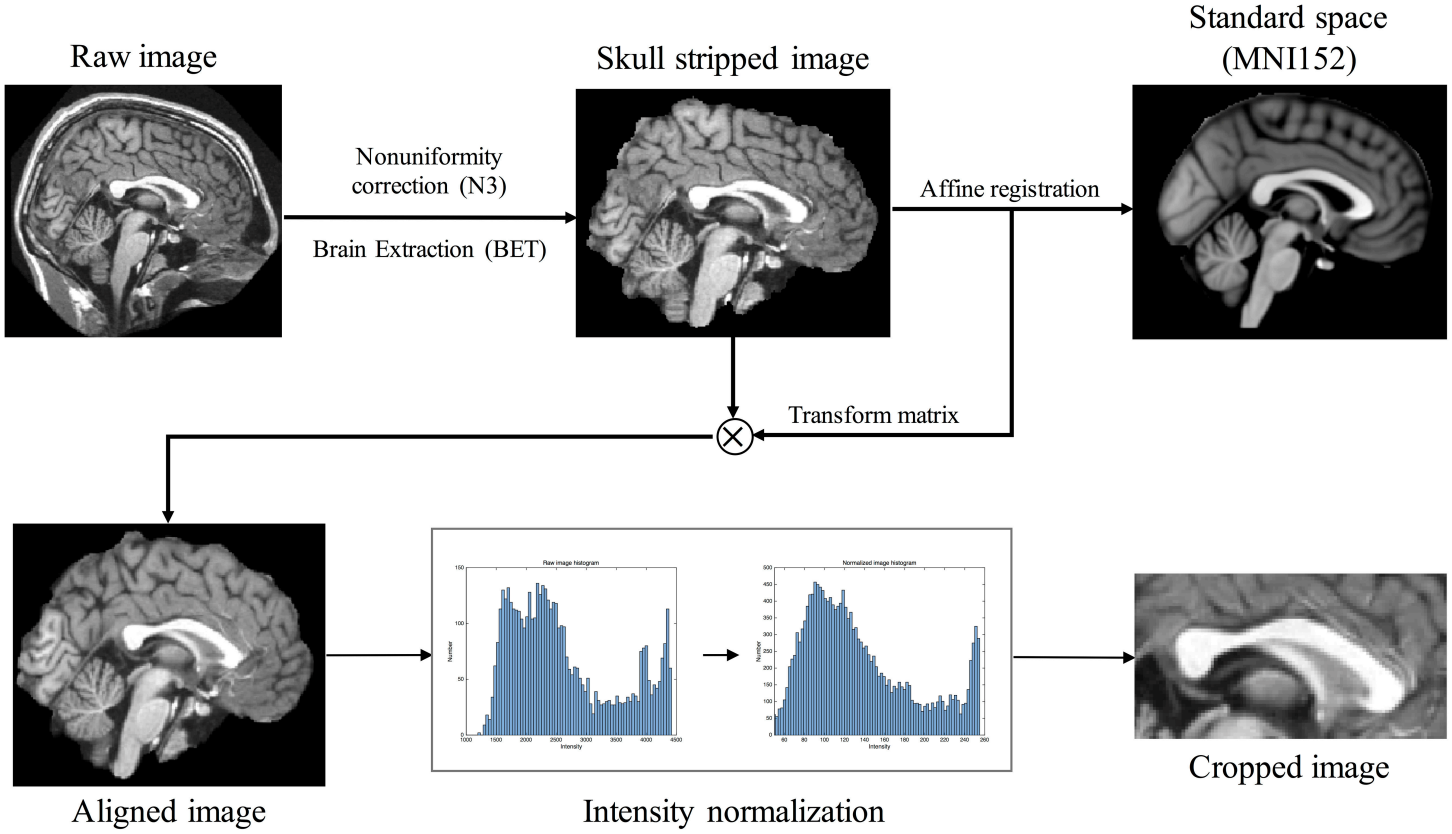
Raw image pre-processing flow chart consisting of the following steps: (1) intensity non-uniformity correction; (2) brain extraction; (3) aligning the image with standard space MNI152 through affine registration; (4) intensity normalization ranging from 0 to 255 using 2nd to 98th percentiles intensities in each image histogram; (5) image cropping in midsagittal plane.

All T1-weighted MR images were passed through pre-processing (Figure 1). Intensity non-uniformity correction (Sled et al., 1998) was followed by brain extraction tool (BET) to extract brain region (Smith, 2002). The images were, then, aligned to the MNI152 average template using an affine transformation of FLIRT of FSL tool (Jenkinson and Smith, 2001; Jenkinson et al., 2002) and the intensities ranged from 2nd to 98th percentiles were normalized to 0 and 255. Since the images were aligned to the standard space, the median slice of x-axis was extracted as MSP and a rectangular region containing CC was cropped. An expert (Gilsoon Park) manually delineated the CC as the gold standard to evaluate the proposed method and the atlas to segment target image. We manually delineated CC according to the protocol suggested by Luders et al. (2005) and Firat et al. (2014). CC in MSP shows clear regions which are splenium, isthmus, posterior body, anterior body, and anterior third. The gold standard images were manually drawn along these regions. We evaluated manually delineated image by measuring intraclass reliability coefficient (ICC). ICC of 0.9931 was obtained for inter-rater reliability (2 raters: Gilsoon Park and Kichang Kwak) and 0.9940 for intra-rater reliability (3 repeats) with 5 Images are randomly selected in each data set.

### Prior information estimated from multi-atlas voting

**Figure 2:**
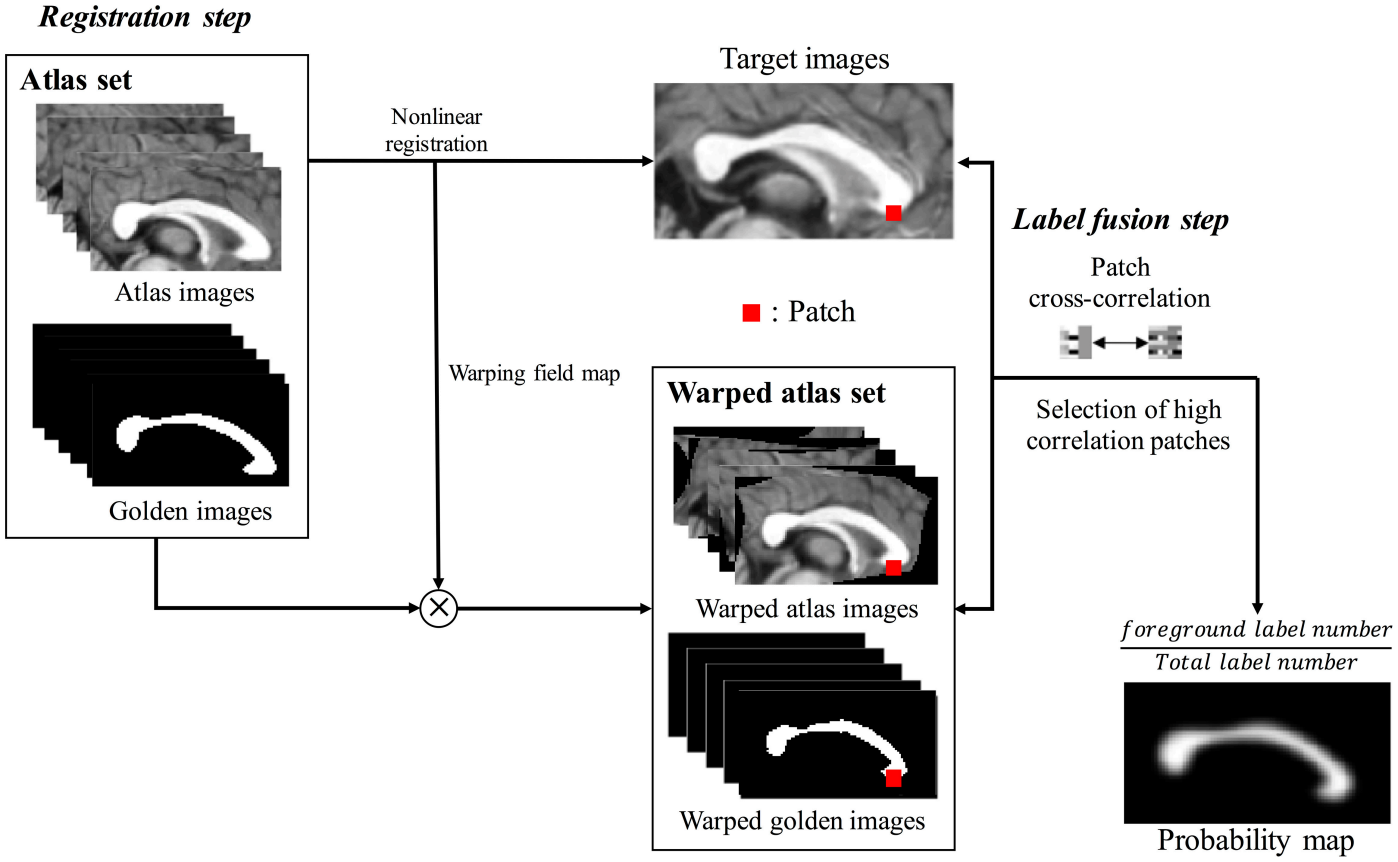
The flow chart of building prior information estimated from multi-atlas voting (PIEMV). This process consists of registration step and label fusion step. In registration step, all cropped atlas images were registered to cropped target image, generating warping field map. Using warping field map, the atlas images and golden images were aligned to a target image. Next label fusion step, the cross-correlation values of patches between target image and aligned atlas images are calculated. Then, the patches with high correlation are selected, generating probability map containing prior information of overall shape and location of corpus callosum of target image.

Multi-atlas voting based segmentation consists of registration and label fusion steps (Figure 2). First, all cropped atlas images were non-linearly registered to a target image, resulting in generation of warping information in each image. Non-linear registration was performed using Automated Image Registration tool (AIR 5.3.0; http://air.bmap.ucla.edu/AIR5/) (Woods et al., 1998). The atlas images and their corresponding label images were aligned to a target image using warping information. The non-linear registration for aligning atlas set to target image is the typical step in multi-atlas voting based segmentation (Aljabar et al., 2009; Wang et al., 2013; Asman et al., 2015). The spatial agreement of the atlas image with the target image is greatly increased due to the non-linear registration, which improves the results of the label fusion. Second, we compared local cross-correlation values between patches, which is a window containing both target voxel and its neighbor voxels on each target voxel location, from aligned atlas images and the target image to choose the subset of atlases. The probability map, overlapping the label images of the subset, was acquired to contain the information of shape and location of CC, which was used as prior information (PIEMV) in the proposed Bayesian framework.

### Likelihood estimated from sparse representation error

**Figure 3:**
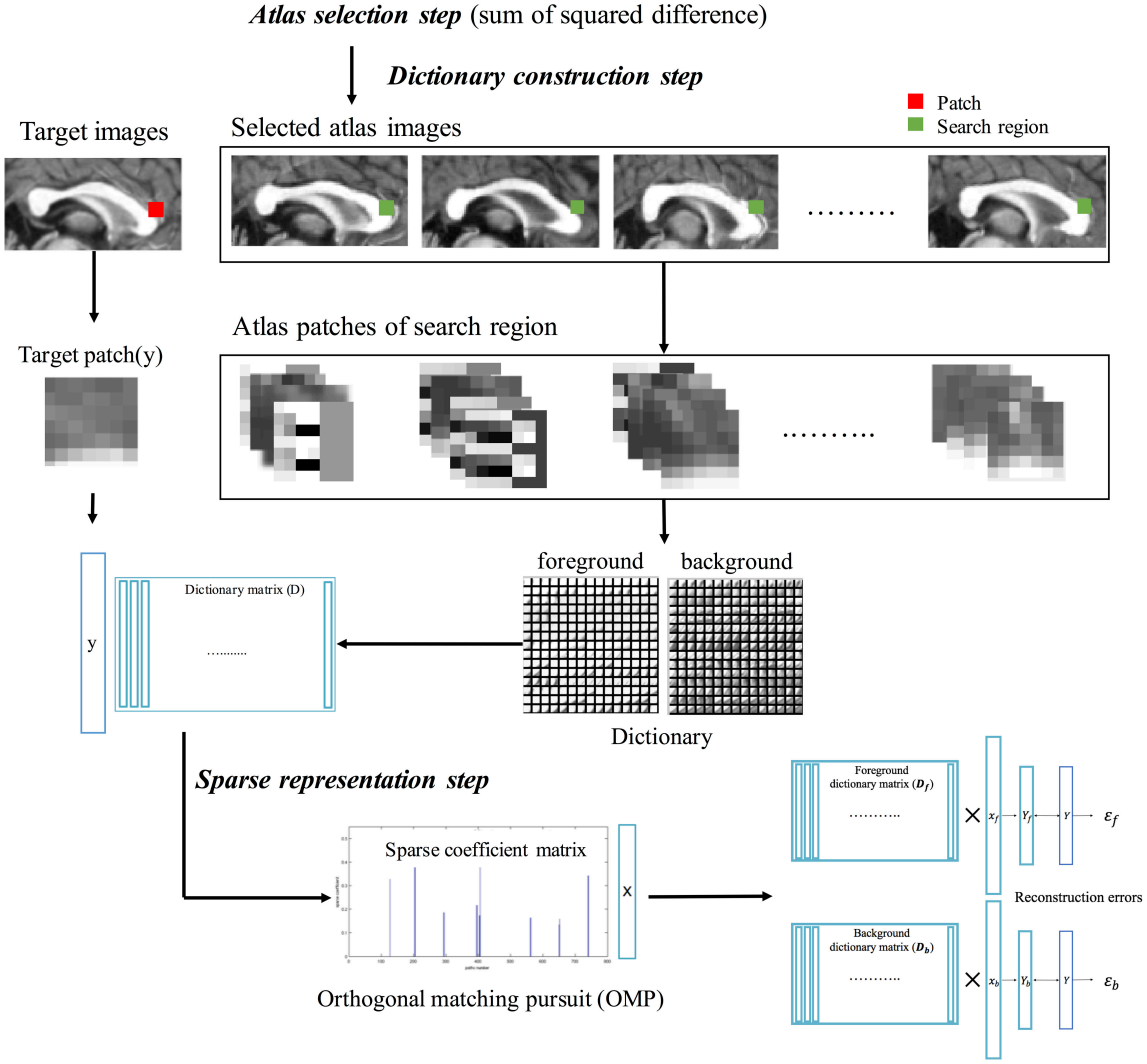
The calculation process of sparse representation error, consisting of atlas selection step, dictionary construction step, and sparse representation step. First, atlas images similar to target image are selected via sum of squared intensity difference. Second, patches are extracted from search region in selected atlas images, constructing atlas patch library. Using label information of atlas golden images, foreground dictionary and background dictionary are generated. Target patch also is extracted from target image. Finally, the target patch is sparsely represented with each patch extracted from the foreground and background dictionaries using orthogonal matching pursuit (OMP) algorithm. The two type reconstructed patches of target patch are used to calculate sparse representation errors.

Estimation of sparse representation error was like the method proposed by Mairal et al., but different in the construction of the patch as atom for the dictionary. The patch consisted of image gradient magnitude and direction as well as image intensity because of obvious shape boundary of CC. The LESRE was used in Bayesian inference. We constructed two types of patches: intensity information as only (Old LESRE) and combining gradient information and intensity information together (LESRE) to evaluated how much performance improve by adding gradient information to patch. The process of extracting sparse representation error has the following three steps (Figure 3): (1) the atlas selection step, which selected the subset of the atlases using similarity measurement between atlases and target image; (2) the dictionary construction step, which constructed the dictionary consist sing of the patches extracted from the search region of the selected atlases and extracted the target patch on target image for labeling a target voxel; (3) the sparse representation step, which sparsely represented the target patch as a linear combination of the patches extracted from dictionary using orthogonal matching pursuit (OMP) algorithm.

The atlas selection depending on target image was carried out based on similarity measurement, which was implemented as sum of squared intensity difference in this paper. This selection step reduces computational time and error resulting from naturally individual variation. A search region *A*_*i*_ was defined in each selected atlas image. All patches on the search region were used to construct foreground (i.e., CC) dictionary *D*_*f*_ if the center voxel of the patch is labeled foreground, or background dictionary *D*_*b*_ otherwise. Finally, we proposed the LESRE based on Wright et al. (2009). The target patch *y* was represented approximately by the patches *d*_*li*_ of dictionary:

(1) $$\begin{array}{r} y={x_{l1}d}_{l1}+{x_{l2}d}_{l2}+\ldots {x_{\ln}d}_{\ln}\;, \end{array}$$

where *l* is the label (i.e., *l* ∈ {*f*(*foreground*), *b*(*background*)}), *n* is the column vector number (i.e., patch number) in each dictionary *D*_*l*_, *x*_ln_ is weight coefficients. Since sparse representation demand that representation is sparsity, most of weight coefficients *x*_ln_ will be zero. Let $X_{l}=\;\left[x_{l1},\;x_{l2},\;\ldots ,\;x_{\ln}\right]\in R^{n}$, which is sparse coefficient matrix, then the target patch *y* is also represented as follow:

(2) $$\begin{array}{r} y={X_{l}D}_{l}+\varepsilon_{l}, \end{array}$$

where ε_*l*_ is reconstruction error that is the difference between target patch and sparse representation. The sparse coefficient matrix *X*_*l*_ can be computed by solving the following L1-norm minimization equation:

(3) $$\begin{array}{r} {\hat{X}}_{l}\;=\;\underset{Xl}{\text{a}rg\text{min}}||X_{l}|{|}_{1\;}\text{subject to }||y-X_{l}D_{l}|{|}_{2}^{2}\leq \varepsilon_{l}\;, \end{array}$$

Equation (3) consist of L1-norm minimization and L2-norm minimization which indicate sparsity level designated user and searching appropriate column vector representing target patch *y* as linear combination. This equation can be efficiently solved by OMP algorithm as described in Tropp and Gilbert (2007), which can safely recover signal with sparsity non-zero coefficients. We selected a column vector *d*_ln_ with max projection value on residue *r*_*k*_ (initial residue *r*_0_ = *y*) from *D*_*l*_ at each iteration *k* and obtained a new sparse representation signal consisting of *X*_*lk*_ = {ẋ_*l*1_, ẋ_*l*2_, …, ẋ_*lk*_} and $D_{lk}=\;\dot{\{d_{l1}},\;\dot{d_{l2}},\;\ldots ,\;\dot{d_{lk}\}}$ by solving a least squares problems arg ||*y* − *X*_*lk*_*D*_*lk*_||_2_, where ẋ_*lk*_ is sparse weight coefficient corresponding to selected column vector $\dot{d_{lk}}$. Finally, we updated residue and dictionary matrix by excluding $\dot{d_{lk}}$. This procedure was iterated until terminating condition given user. The final residue was the reconstruction error ε_*l*_ and played a key role in LESRE. We compared the reconstruction errors generated from each class, that is, the label of the target patch *y* based on LESRE only was given as:

(4) $$\begin{array}{r} L\left(y\right)=arg\underset{l}{\text{min}}\varepsilon_{l}\left(y\right)\;, \end{array}$$

The label function *L* of *y* patch assigned foreground or background according to minimum ε_*l*_ of *y* patch over all classes. Note that the *l* had only two labels consisting of foreground belonging to CC and background in this study.

### Bayesian inference based segmentation

The proposed method, Bayesian inference based segmentation (BIbS), combining prior information estimated from multi-atlas voting (PIEMV) containing overall structure information and LESRE containing detailed structure information in the Bayesian framework. Given target patch *y*_*i*_ at *i th* voxel, the Bayesian inference formulation of *y*_*i*_ patch is as follow:

(5) $$\begin{array}{r} p\left(c_{l}|y_{i}\right)=\;\frac{p\left(y_{i}|c_{l}\right)p\left(c_{l}\right)}{p\left(y_{i}\right)}\;\left(l=1,2,\;\ldots ,\;C,\;i=1,2,\;\ldots ,\;n\right), \end{array}$$

where *p*(*c*_*l*_|*y*_*i*_), posterior probability, is the probability of belonging to class *c*_*l*_ via *y*_*i*_, *C* is class or label number, *n* is all voxel number in cropped image, *p*(*y*_*i*_|*c*_*l*_), likelihood, is the probability of observing *y*_*i*_ in class-conditional probability distribution according to *c*_*l*_, *p*(*c*_*l*_), prior probability, is the probability of observing *c*_*l*_ reflecting our knowledge of state of nature *c*_*l*_, and *p*(*y*_*i*_) is evidence which is used to normalize the product of likelihood and prior probability, which does not affect determining the relative probabilities of each class. The class that maximizes the posterior probability is assigned to the target patch *y*_*i*_.

The PIEMV was used as the prior probability in Bayesian inference since it includes pre-defined anatomical knowledge of shape and location of CC. Therefore, the prior probability of observing a class at given patch *y*_*i*_ was:

(6) $$\begin{array}{r} p\left(c_{l}\right)=\frac{\sum_{a=1}^{N}\delta \left(L_{a}\left(i\right),\;l\right)}{N}, \end{array}$$

where *N* is selected atlas number of forming probability map, δ (,) is the Kronecker delta function, and *L*_*a*_(*i*) is the label from the labeled image of *a th* atlas image at *i th* voxel. The reconstruction error of LESRE was used as class-conditional probability distribution model using gamma distribution. The gamma distribution reflects the following two properties of the distribution of the error ratio (i.e., foreground error rate/background error rate): (1) the smaller the error ratio generated from label dictionaries, the higher the likelihood of belonging to the label; (2) the frequency of the error ratio close to zero sharply decreases.

(7) $$\begin{array}{rcl} p\left(y_{i}|c_{l}\right) & = & p\left(\varepsilon_{i}|D_{l}\right).\\ p\left(\varepsilon_{i}|D_{l}\right) & = & \frac{{\varepsilon_{i}}^{\alpha_{l}-1}e^{{\frac{\varepsilon_{i}}{\beta_{l}}}_{\;}}}{\Gamma \left(\alpha_{l}\right)\;\beta_{l}^{\alpha_{l}}}, \end{array}$$

where ε_*i*_ is reconstruction error ratio at *i th* voxel, resulting from sparse representation based on dictionary *D*_*l*_ consisting of error ratios labeled class *l* and α_*l*_ is shape parameter and β_*l*_ is scale parameter in each class. The α_*l*_ and β_*l*_ are obtained by reconstruction error ratio set of atlas images we know label. Finally, we labeled the target voxel through comparison of posterior probability:

(8) $$\begin{array}{rcl} p\left(y_{i}|c_{l}\right)p\left(c_{l}\right) & = & \frac{{\varepsilon_{i}}^{\alpha_{l}-1}e^{{\frac{\varepsilon_{i}}{\beta_{l}}}_{\;}}\sum_{a}^{N}\delta \left(L_{a}\left(i\right),\;l\right)}{\Gamma \left(\alpha_{l}\right)\;\beta_{l}^{\alpha_{l}}\;N}\;\left(\varepsilon_{i}\;\geq 0\right).\\ L_{p}\left(y_{i}\right) & = & \;\{\;\begin{array}{c} foreground\;\frac{{\varepsilon_{i}}^{\alpha_{f}-1}e^{{\frac{\varepsilon_{i}}{\beta_{f}}}_{\;}}\Gamma \left(\alpha_{b}\right)\;\beta_{b}^{\alpha_{b}}}{{\varepsilon_{i}}^{\alpha_{b}-1}e^{{\frac{\varepsilon_{i}}{\beta_{b}}}_{\;}}\Gamma \left(\alpha_{f}\right)\;\beta_{f}^{\alpha_{f}}}\;\geq \;\frac{\sum_{a}^{N}\delta \left(L_{a}\left(i\right),\;b\right)}{\sum_{a}^{N}\delta \left(L_{a}\left(i\right),\;f\right)}\;\\ background\;\frac{{\varepsilon_{i}}^{\alpha_{f}-1}e^{{\frac{\varepsilon_{i}}{\beta_{f}}}_{\;}}\Gamma \left(\alpha_{b}\right)\;\beta_{b}^{\alpha_{b}}}{{\varepsilon_{i}}^{\alpha_{b}-1}e^{{\frac{\varepsilon_{i}}{\beta_{b}}}_{\;}}\Gamma \left(\alpha_{f}\right)\;\beta_{f}^{\alpha_{f}}}\;<\;\frac{\sum_{a}^{N}\delta \left(L_{a}\left(i\right),\;b\right)}{\sum_{a}^{N}\delta \left(L_{a}\left(i\right),\;f\right)} \end{array}\;, \end{array}$$

where *L*_*p*_ of *y*_*i*_ patch is labeling function using Bayesian inference at *i th* voxel. The left and right side of comparison consist of above-mentioned likelihood and prior probability each.

### Validation

We used the Dice index (DI), a quantitative measure of the spatial agreement, to evaluate the segmentation accuracy of the proposed method as follows:

(9) $$\begin{array}{r} \text{DI}\left(\text{A},\text{ B}\right)=\frac{2|A\cap B|}{\left|A|+|B\right|}\times 100\;\left(\%\right), \end{array}$$

where | · | is the number of voxels consisting of positive region in binary segmentation image, and |*A*∩*B*| is the number of voxels of common region between *A* and *B* binary segmentation image. The binary segmentation images for using DI evaluation are golden standard image made by manual delineation and segmentation result of proposed method. The Dice index range from 0 to 100 percentage, and the value close to 100%indicates the better segmentation performance.

We further evaluated the JLF method (Wang and Yushkevich, 2013; Wang et al., 2013) for CC segmentation and compared our method (BIbS) with JLF in a quantitative and qualitative aspects. JLF improved weighted voting of label fusion step in multi-atlas voting method. The weighed voting of JLF consider pairwise dependency between atlases as explicitly modeled the joint probability of two atlases making a labeling error at a voxel as well as minimizing the total expectation of labeling error (Wang et al., 2013). JLF was conducted using open-source (https://www.nitrc.org/projects/picsl_malf; version 1.3).

The leave-one-out cross validation was performed on each data set to compare the performance of each segmentation method. In OASIS data set, one subject was selected as target image and the other 99 subjects were used as atlas set, and this process was repeated 100 times. In SMC data set, one subject was selected as target image and the other 46 subjects were used as atlas set, and this process was repeated 47 times. A paired *t*-test was conducted to analyze statistical differences in the Dice index distribution resulting from each method at each data set.

## Results

### Comparison of methods

**Figure 4:**
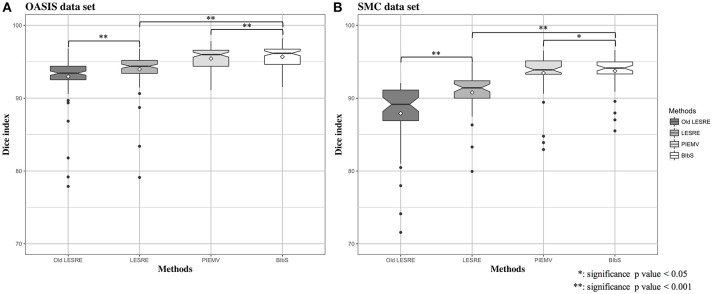
Box plots for Dice index of segmentation results in **(A)** OASIS data set and **(B)** SMC data set. In OASIS data set, the results of mean Dice index are that PIEMV is 95.44 ± 1.58 (%), old LESRE is 92.96 ± 2.77 (%), LESRE 93.99 ± 2.30 (%), and BIbS is 95.68 ± 1.54 (%). In SMC data set, the results of mean Dice index are that PIEMV is 93.45 ± 2.93 (%), old LESRE is 87.91 ± 4.67 (%), LESRE is 90.77 ± 2.70 (%), and BIbS is 93.74 ± 2.30 (%). The results indicate two facts that BIbS consisting of multi-atlas voting as prior information and sparse representation error as likelihood has better segmentation performance than the other method with one infromation (i.e., PIEMV and LESRE) and the LESRE with intensity information and gradient information has good segmentation performance than old LESRE with only intensity information.

We used leave-one-out cross validation to evaluate segmentation performance, where one subject was selected as target image and the other subjects as atlas image. All method was conducted on each optimal parameter set. The segmentation results of JLF was generated from optimal four primary parameters which are the radius of the image patch (*r*_*p*_), the radius of the search neighborhood (*r*_*s*_), the model parameter for transferring image similarity measures into atlas dependencies (β), and the weight of the conditioning identity matrix added to dependency matrix (α). We set the parameter set according to Wang and Yushkevich (2013), which were *r*_*p*_ = 1, *r*_*s*_ = 3, β = 2, and α = 0.1 in OASIS data set and *r*_*p*_ = 2, *r*_*s*_ = 3, β = 2.5, and α = 0.1 in SMC data set. The parameters of PIEMV were the number of atlases and path size. The optimal number of atlases were determined 10 in OASIS and 5 in SMC data set respectively and the optimal path size of both data sets 7 × 7 in PIEMV (Figure S1). The parameters of LESRE were the number of atlases, search region size and path size. The optimal number of atlases was determined 25 and 15, the optimal search region size 7 × 7 and 7 × 7, and the optimal patch size 13 × 13 and 9 × 9 in OASIS and SMC data sets respectively in LESRE (Figure S2). Table 2 shows segmentation results for the mean Dice index and standard deviation in each data set. The table compared the results of the suggested methods with the other methods. In OASIS data set, the results of mean Dice index are that JLF is 96.38 ± 1.78, Old LESRE is 92.96 ± 2.77 (%), LESRE is 94.09 ± 1.82 (%), PIEMV is 95.44 ± 1.58 (%), and BIbS is 95.80 ± 1.52 (%). In SMC data set, the results of mean Dice index are that JLF is 94.99 ± 1.89, Old LESRE is 88.43 ± 4.17 (%), LESRE is 90.71 ± 2.83 (%), PIEMV is 93.4 ± 2.93 (%), and BIbS is 93.72 ± 2.58 (%). Figure 4 shows box plots for the Dice index of each method excepted JLF method in both data sets. The black lines and diamond shapes in box plots are median points and mean points respectively. The Figure 4 indicated BIbS has statistically significant better segmentation performance (*p*-value in OASIS data set: 7.77e-04, *p*-value in SMC data set: 0.0436) than both LESRE and PIEMV in both data sets and LESRE has statistically significant better segmentation performance than old LESRE. The segmentation results resulting from SMC data set have the worse segmentation performance than OASIS data set because of variation of CC induced heavy drinkers and old age. The average computation time per image of JLF, PIEMV, LESRE, and BIbS in each optimal parameter set were 10, 68, 124, and 141 s each on a Linux workstation with 2.4 GHz clock speed. JLF was implemented in C language and the other methods were implemented in MATLAB 9.1.0 excepted non-linear registration.

**Table 2:** Mean Dice index and standard deviation of segmentation results in OASIS data set and SMC data set.

	**Dice index**			
	**OASIS data set**		**SMC data set**	
	**Mean**	**Standard deviation**	**Mean**	**Standard deviation**
JLF	96.38	1.78	94.99	1.89
Old LESRE	92.96	2.77	87.91	4.67
LESRE	93.99	2.30	90.77	2.70
PIEMV	95.44	1.58	93.45	2.93
BIbS	95.68	1.54	93.74	2.30

### The effect of parameters of BIbS on segmentation performance

**Figure 5:**
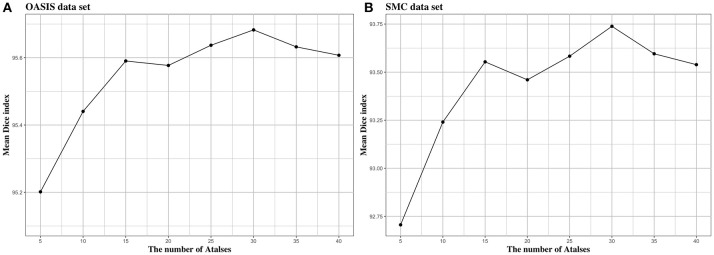
The trend of mean Dice index of BIbS results depending on the number of atlases in **(A)** OASIS data set and **(B)** SMC data set. The segmentation performance was evaluated by adjusting the number of atlases from 5 to 40 by 5 increments. The other parameters, search region size, and patch size, are fixed. In both OASIS and SMC data set, the search region size was fixed at 7 × 7 and the patch size was fixed at 13 × 13. The results show that when the number of atlases exceeds a certain number (In both OASIS and SMC data set: 30), the segmentation performance is saturated.

**Figure 6:**
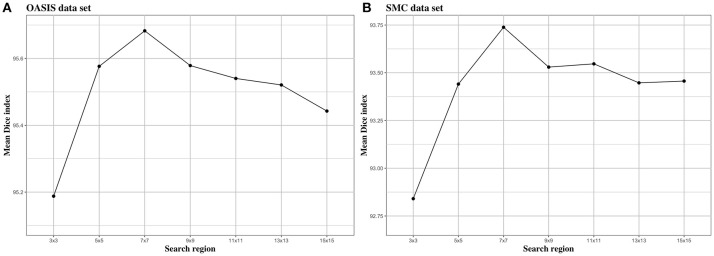
The trend of mean Dice index of BIbS results depending on search region. The segmentation performance was evaluated by adjusting the size of search region from 3 × 3 to 15 × 15 by 2 × 2 increments. The other parameters, atlas selection number and patch size, are fixed. In both **(A)** OASIS data set and **(B)** SMC data set, the atlas selection number was fixed at 30 and the patch size was fixed at 13 × 13. The results show that when the search region reaches a certain size [In both **(A)** OASIS data set and **(B)** SMC data set: 7 × 7], the segmentation performance is saturated or decreased.

**Figure 7:**
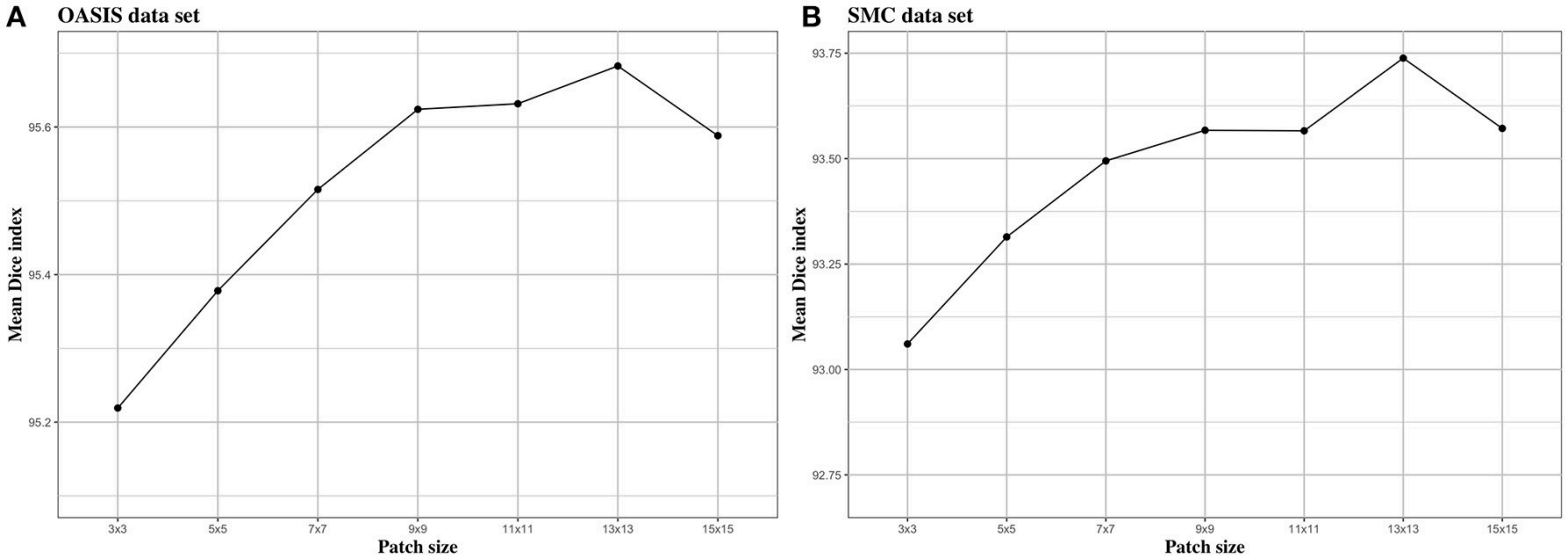
The trend of mean Dice index of BIbS results depending on patch size. The segmentation performance was evaluated by adjusting the size of patch from 3 × 3 to 15 × 15 by 2 increments. The other parameters, atlas selection number and search region size, are fixed. In both **(A)** OASIS data set and **(B)** SMC data set, the atlas selection number was fixed at 30 and the search region size was fixed at 7 × 7. The results show that when the patch reaches a certain size [In **(A)** OASIS data set and **(B)** SMC data set: 13 × 13], the segmentation performance is saturated.

We investigated the effect of parameters in BIbS, which are the number of atlases, search region, and patch size, on segmentation performance. Figures 5–7 show the relationship between segmentation performance of BIbS (mean Dice index) and the parameters in BIbS. The optimal values of parameters are as follow in both OASIS data set and SMC data set: (1) the number of atlases: 30; (2) search region: 7 × 7; (3) patch size: 13 × 13. In each evaluation, we observed the BIbS performance trend according to target parameter, and the other parameters fixed at optimal values. First, we evaluated the BIbS performance trend according to the number of atlases ranging from 5 to 40 by 5 increments on OASIS data set and SMC data set (Figure 5). The optimal number of atlases with maximum segmentation performance is 30 in both OASIS data set and SMC data set. After the optimal point, the segmentation performance of BIbS decreased. The width of change of mean Dice index about the number of atlases is 0.48 and 1.03 in OASIS data set and SMC data set, respectively. Second, we investigated the effect of search region on BIbS performance. The search region was changed from 3 × 3 to 15 × 15 by 2 × 2 increments. The BIbS performance trend depending on search region was reported in Figure 6. The BIbS performance reached saturation point at 7 × 7 search region in both data sets. The width of change of mean Dice index about the search region is 0.49 and 0.90 in OASIS data set and SMC data set, respectively. Finally, the BIbS performance trend according to patch size was studied on OASIS data set and SMC data set. The patch size was changed from 3 × 3 to 15 × 15 by 2 × 2 increments. Figure 7 shows that the maximum of the BIbS performance according to patch size is 13 × 13 in both OASIS data set and SMC data set. After the maximum point, the segmentation performance of BIbS decreased. The width of change of mean Dice index about patch size is 0.46 and 0.68 in OASIS data set and SMC data set, respectively.

### Images of segmentation results of proposed method

**Figure 8:**
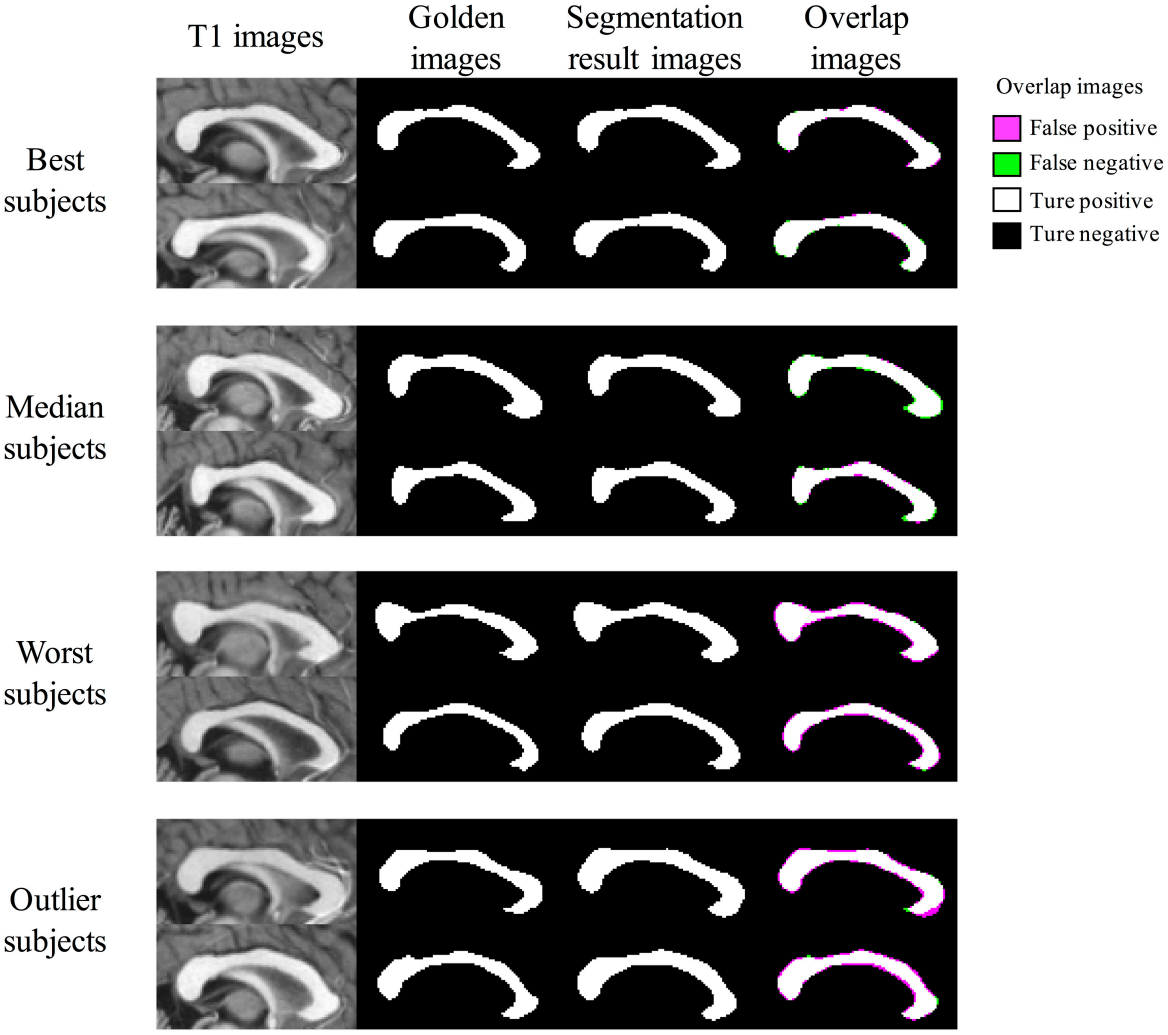
The Segmentation result images of BIbS method in OASIS data set. The images are divided into best subjects, median subjects, worst subjects, and outlier subjects according to Dice index. The overlap images of outlier subjects and worst subjects have many false positive because of dilated segmentation results. The most of false positives result from inter variability problems of manual segmentation because of as follow: (1) the segmentation results of all the subjects are robust, but CCs of golden images of the worst subjects and outlier subjects are relatively thin compared to best subjects and median subjects; (2) the false positive is generated uniformly along the boundary line of the segmentation results in the worst subjects and the outlier subjects.

Figure 8 shows segmentation results of BIbS method in OASIS data set, which consist of best subjects, median subjects, worst subjects, and outlier subjects based on Dice index. From overlap images of outlier subjects and worst subjects, we observed that many false positive occurred because of dilated segmentation results. The top subject of outlier subjects has the noise of bright intensity in the genu of CC, resulting in many false positive. The other false positive shows inter variability problem of manual segmentation rather than showing the problem of the proposed method. In fact, in Figure 8, the segmentation results of all the subjects are robust, but CCs of golden images of the worst subjects and outlier subjects are relatively thin compared to the other subjects. Also, it strongly indicates the inter variability problem of manual segmentation that the most of false positives is generated uniformly along the boundary line of segmentation results in the outlier subjects and worst subjects. Nevertheless, The Dice index of the worst subjects and outlier subjects exceeded 91 and 92%, respectively. In addition, the fornix and noise with intensity like CC were not detected as false positives in segmentation results of other subjects except the top subject of outlier subjects.

## Discussion

### Comparison of methods

In this paper, we introduced a novel method for the segmentation of CC. From Table 2 and Figure 4, we observed that BIbS had better segmentation performance than both LESRE and PIEMV. It is mean that PIEMV and LESRE are effectively combined on Bayesian inference by improving strengths and weakness of each methods. From comparison of between old LESRE and LESRE, we know that the gradient information significantly improves segmentation performance, since the information reinforce difference between foreground and background for sparse representation. The segmentation performance of SMC data set is relatively lower than OASIS data set because of SMC data set features consisting of elders and heavy drinkers, resulting in variation of CC. But, despite variation of CC the proposed method shows good segmentation performance with 93.74% of mean Dice index.

**Figure 9:**
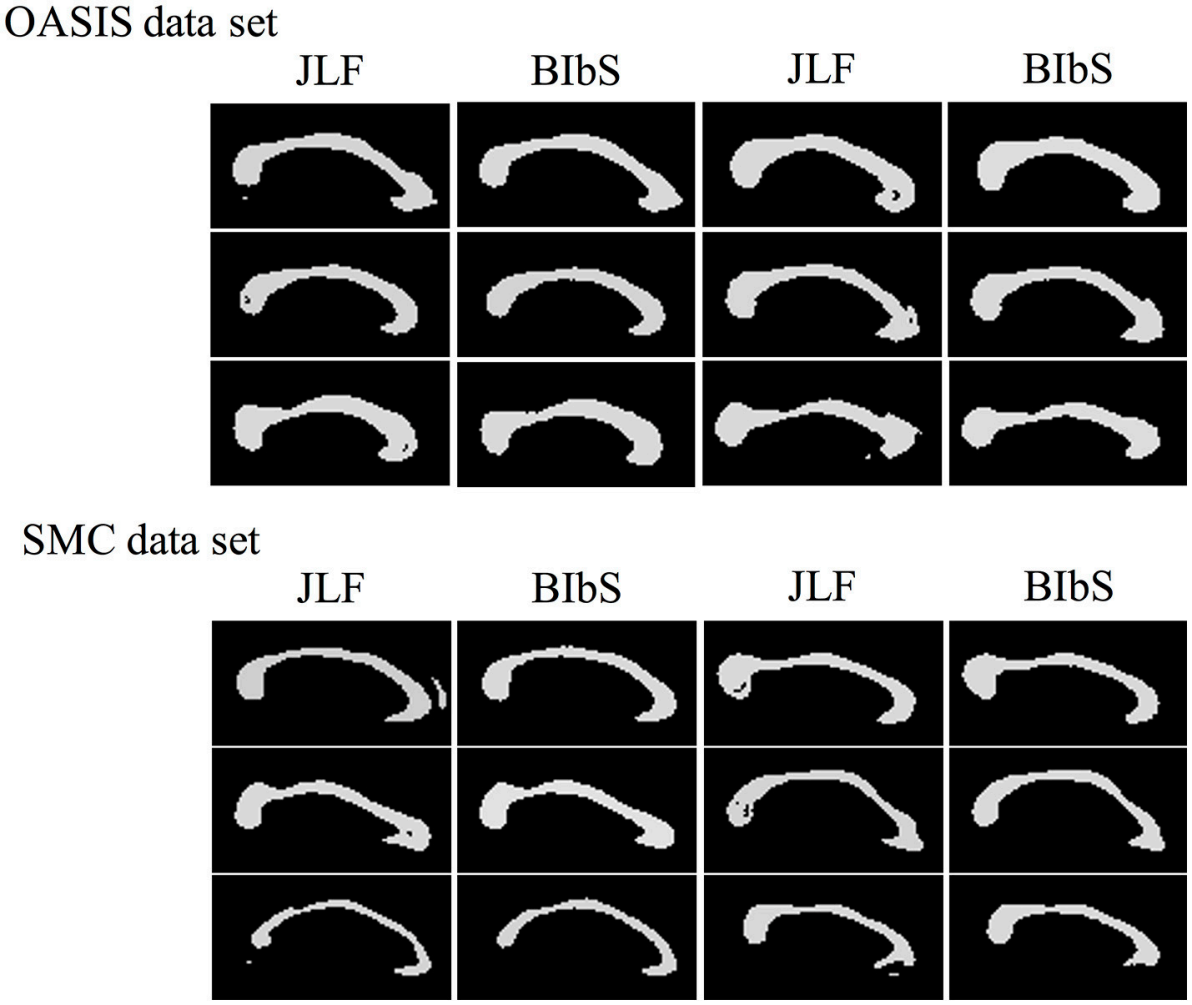
The Segmentation result images of our method (BIbS) and joint label fusion (JLF) method in OASIS data set and SMC data set. JLF creates segmentation image of CC having holes or small region far from CC of ground truth, but these results were disappeared in BIbS method.

While JLF had a higher Dice index of about 0.5–1% than BIbS in quantitative aspect, JLF had holes or small regions far from CC that rarely occur in BIbS, which were more frequent and worse in the SMC data set having large CC variation in qualitative aspect (Figure 9). It is important to extract CC as one closed curve since it is generally used as seed for fractional anisotropy (FA) analysis and fiber extraction. Table 3 showed the number of subjects belonging to each value of connected components and Euler number of each method at each data set. If the connected component is not 1, it indicates that the result has a small region far from CC, and if the Euler number is not 1, it indicates that the result has a hole or small region exists. BIbS does not have holes or small regions except only one subject in OASIS data set, but JLF has holes or small regions in many subjects, which are more frequent and worse in SMC data set having large CC variation. The qualitative results of JLF could be improved if proper post-processing is applied or Bayesian framework is adapted to use JLF as prior information. While JLF showed a slightly better quantitative result, our proposed method based on Bayesian framework could be improved by adopting state-of-the-art multi-atlas voting like JLF as prior information.

**Table 3:** The number of subjects belonging to each value of connected components and Euler number of each method at each data set.

		**Connected components**		**Euler number**			
		**1**	**2**	**−1**	**0**	**1**	**2**
OAIS data set (100 subjects)	BIbS	99	1	0	0	99	1
	JLF	96	4	2	3	91	4
SMC data set (47 subjects)	BIbS	47	0	0	0	47	0
	JLF	44	3	4	1	39	3

*If the connected component is not 1, it indicates that the result has a small region far from CC, and if the Euler number is not 1, it indicates that the result has a hole or the small region exists. The results of BIbS method have not the hole or the small region except only one subject in OASIS data set, but the results of JLF method have the hole or the small region in many subjects and these results of JLF method are more frequent and worse in SMC data set having large CC variation*.

### The effect of parameters of BIbS on segmentation performance

We examined the effect of BIbS parameters, which are the number of atlases, search region, and patch size, on segmentation performance (i.e., LESRE parameters included BIbS). The optimization parameter of BIbS in both OASIS and SMC data set is that the atlas selection number is 30, search region size is 7 × 7, and patch size is 13 × 13.

Figures 5–7 described the changes of the segmentation performance of the BIbS depending on each parameters control, and show that the search region has the most influence on segmentation performance in OASIS data set. The search region determines how much the neighborhood patches around the location of the target patch in atlases are included in a dictionary for the sparse representation. As the search region is larger, the patches corresponding to the target patch of target image will be extracted from the atlases and included in the dictionary of foreground or background, resulting in the accuracy of a dictionary for sparse representation is improving. Therefore, the search region should be larger than a certain size (i.e., 3 × 3 in this paper) for the properly working of the sparse representation and if the search region exceeds a certain size (i.e., 7 × 7 in this paper), it no longer has a significant influence on segmentation performance. Figure 6 shows that the segmentation performance of BIbS is increased when the search region is more than a certain size, and the BIbS performance is saturated or decreased by increasing search region.

In Figure 5, the number of atlases most influenced BIbS performance in SMC data set. The number of atlases is associated with the dictionary quality to classify foreground and background. As the number of atlases increases, the atlas less like target image is used and the dictionary consist of various patches, which can improve or worsen segmentation performance. If there are the atlases very similar to a target image, adding the patches from other atlases to the dictionary can reduce segmentation performance. In OASIS data set, the number of subjects is larger than SMC data set and the variations of the CCs are smaller than SMC data set because the OASIS data set consist of normal young people. Therefore, there can be the atlases very similar to the target image in the OASIS data set and using the additional atlases can have slightly better segmentation performance. However, if there is a lot of variations between data like SMC data set, adding the various patches of atlases to the dictionary can improve the segmentation performance. For this reason, Figure 5 shows that the influence of the number of atlases in OASIS data set is lower than in SMC data set.

In Figure 7, patch size slightly influenced BIbS performance in both data sets. The patch size is associated with the spatial information of the patch in the dictionary. The large patch size can improve segmentation performance by adding spatial information to patch, assisting more clear distinction between foreground and background, but can also constitute patch containing unwarranted spatial information. Thus, the patch size, like the number of atlases, has an optimal point depending on the characteristics of the data set. Figure 7 shows that the segmentation performance of BIbS in both data set is increased when the patch size is more than a certain size (i.e., 13 × 13 in this paper), and the BIbS performance is decreased by increasing patch size.

## Conclusion

In this paper, we developed a novel method for CC segmentation in MSP, based on Bayesian inference We used reconstruction error resulting from sparse representation as likelihood and probability map generated from multi-atlas voting method as prior information. The segmentation results show that the combination of sparse representation error and multi-atlas voting, based on Bayesian inference, has the significantly better segmentation performance than using each method alone. We also observed that the data set having large CC variation was sensitive to the number of atlases for good segmentation performance. In the future, we will develop a more robust segmentation method about target image having large variation of CC based on Bayesian inference by improving likelihood and prior information and developing framework to effectively search optimal parameters. We will also apply our method to brain structure which is more difficult to segmentation.

## Author contributions

GP and J-ML study concept and design. GP and J-ML drafting of manuscript. GP, KK, and J-ML statistical analysis and interpretation. SS acquisition of samsung medical center dataset.

### Conflict of interest statement

The authors declare that the research was conducted in the absence of any commercial or financial relationships that could be construed as a potential conflict of interest. The reviewer ML and handling Editor declared their shared affiliation.

## Supplementary material

Figure S1 The trend of mean Dice index of PIEMV results depending on the number of atlases and patch size. The segmentation performance was evaluated by adjusting the number of atlases from 5 to 40 by 5 increments and the size of patch from 3 × 3 to 15 × 15 by 2 increments. The other parameters except the test parameter were fixed to the optimal value. The optimal parameter set of PIEMV in OASIS data set was that the number atlases is 10 and the patch size is 7 × 7. The optimal parameter set of PIEMV in SMC data set was that the number atlases is 5 and the patch size is 7 × 7. The results show that the performance of PIEMV in SMC data set having large CC variation was sensitive to the change of the number of atlases and patch size than OASIS data set.

Figure S2 The trend of mean Dice index of LESRE results depending on the number of atlases, search region size, and patch size. The segmentation performance was evaluated by adjusting the number of atlases from 5 to 40 by 5 increments, the size of search region 3 × 3 to 15 × 15 by 2 increments, and the size of patch from 3 × 3 to 15 × 15 by 2 increments. The other parameters except the test parameter were fixed to the optimal value. The optimal parameter set of LESRE in OASIS data set was that the number atlases is 25, the search region size is 7 × 7, and the patch size is 13 × 13. The optimal parameter set of LESRE in SMC data set was that the number atlases is 15, the search region size is 7 × 7, and the patch size is 11 × 11. The change width of performance according to parameter changes is similar in both data sets.
