# Supplementary figures and images for: Automatic Segmentation of Corpus Callosum in Midsagittal Based on Bayesian Inference Consisting of Sparse Representation Error and Multi-Atlas Voting
Source: Front Neurosci. 2018 Sep 11;12:629. doi: 10.3389/fnins.2018.00629 (PMC6142891; doi:10.3389/fnins.2018.00629)

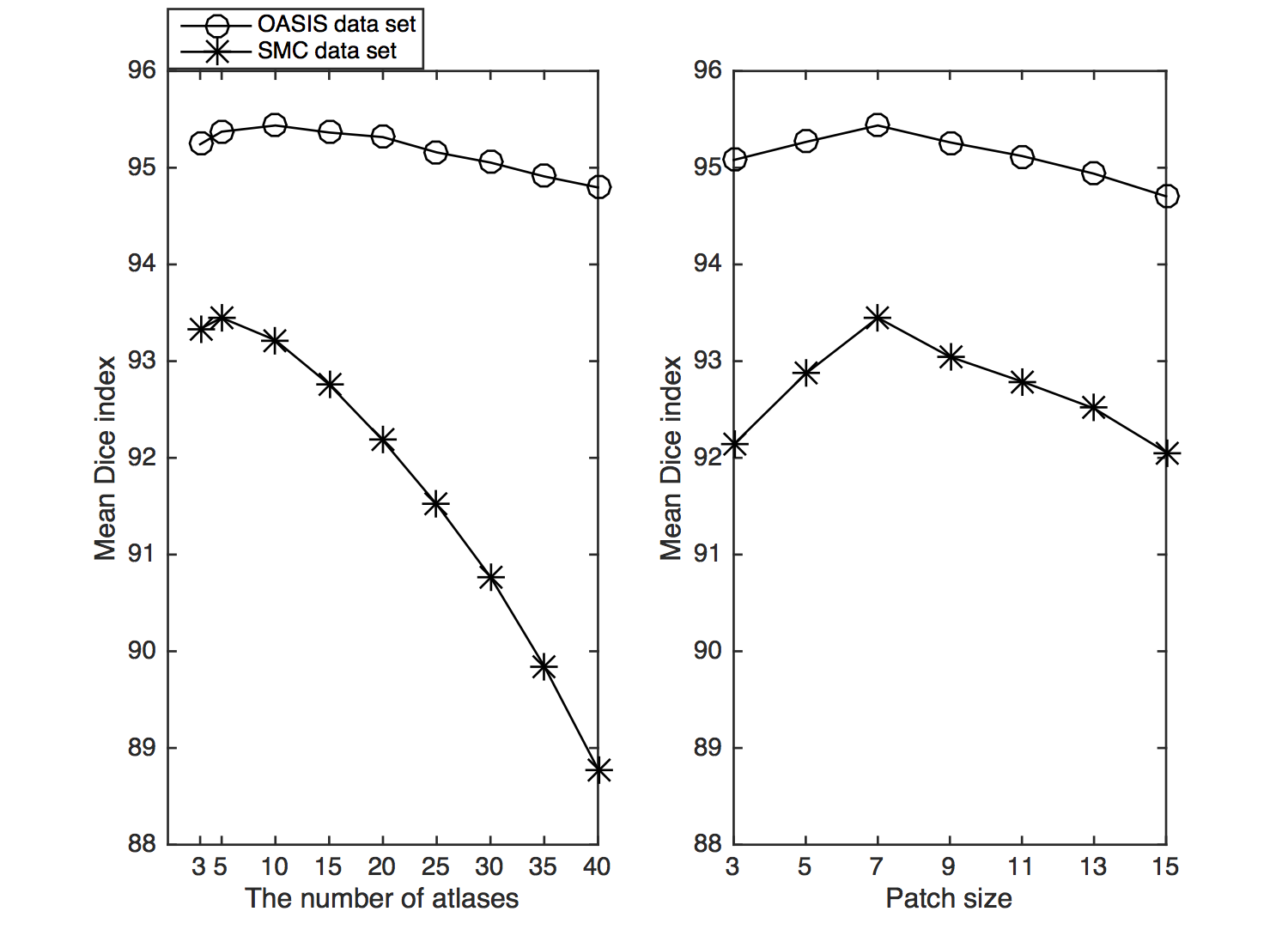

Supplement: Figure S1 — The trend of mean Dice index of PIEMV results depending on the number of atlases and patch size. The segmentation performance was evaluated by adjusting the number of atlases from 5 to 40 by 5 increments and the size of patch from 3 × 3 to 15 × 15 by 2 increments. The other parameters except the test parameter were fixed to the optimal value. The optimal parameter set of PIEMV in OASIS data set was that the number atlases is 10 and the patch size is 7 × 7. The optimal parameter set of PIEMV in SMC data set was that the number atlases is 5 and the patch size is 7 × 7. The results show that the performance of PIEMV in SMC data set having large CC variation was sensitive to the change of the number of atlases and patch size than OASIS data set. [file Image_1.TIFF]

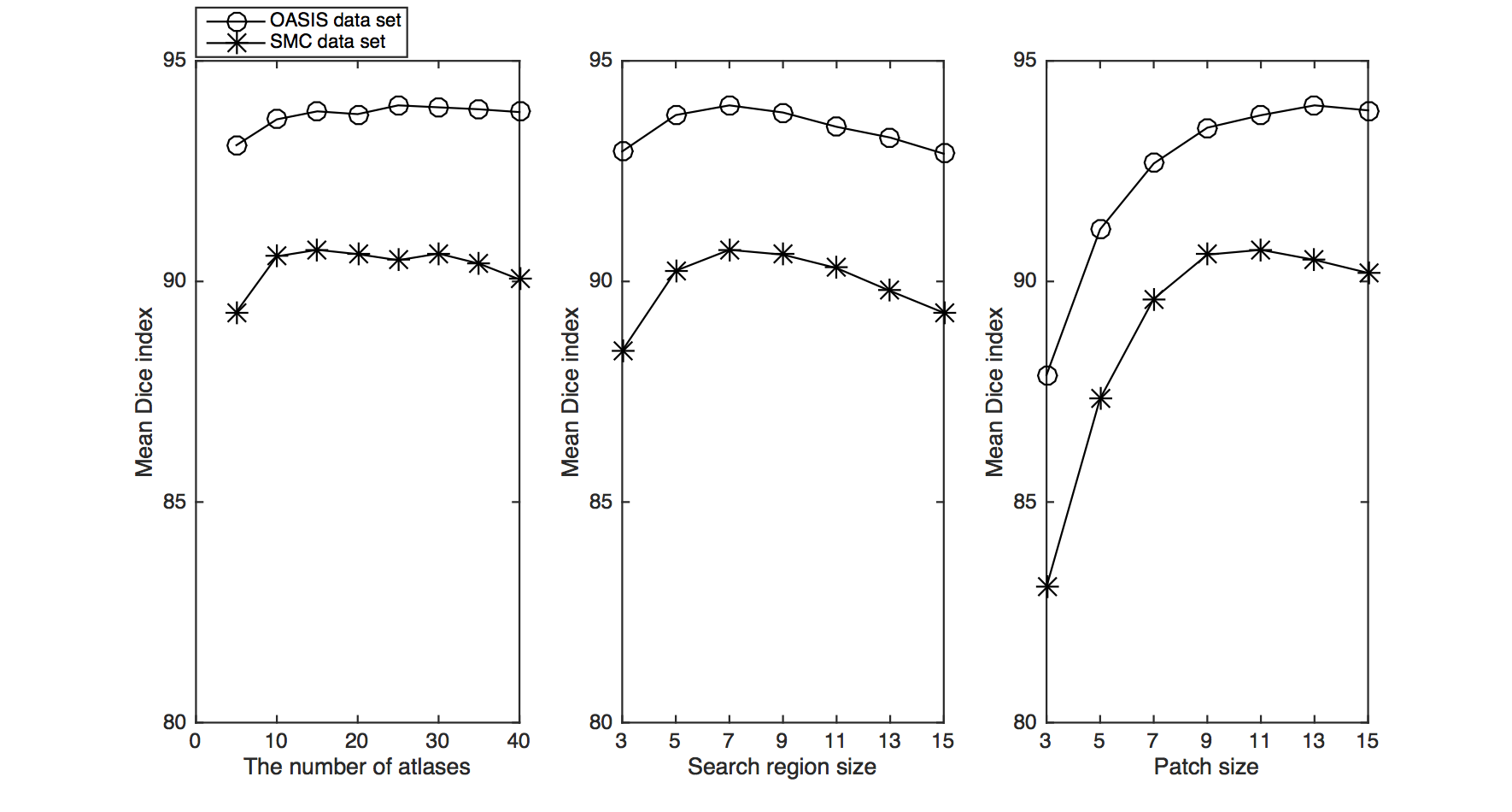

Supplement: Figure S2 — The trend of mean Dice index of LESRE results depending on the number of atlases, search region size, and patch size. The segmentation performance was evaluated by adjusting the number of atlases from 5 to 40 by 5 increments, the size of search region 3 × 3 to 15 × 15 by 2 increments, and the size of patch from 3 × 3 to 15 × 15 by 2 increments. The other parameters except the test parameter were fixed to the optimal value. The optimal parameter set of LESRE in OASIS data set was that the number atlases is 25, the search region size is 7 × 7, and the patch size is 13 × 13. The optimal parameter set of LESRE in SMC data set was that the number atlases is 15, the search region size is 7 × 7, and the patch size is 11 × 11. The change width of performance according to parameter changes is similar in both data sets. [file Image_2.TIFF]
